# Antecedents and Mediators of Academic Satisfaction in Virtual Vocational Training

**DOI:** 10.3390/ejihpe13110174

**Published:** 2023-11-03

**Authors:** José María Figueredo, María del Mar Molero-Jurado, María Francisca Vico-Sánchez, Salvador Hilario Alonso-Delgado, José Javier Rosales-Jiménez, María Concepción Torres-Rojas, Amparo Zorrilla-Lozano, María José Segura-Morillas, María del Carmen Pérez-Fuentes

**Affiliations:** 1 Department of Education of the Andalusian Regional Government, IES Alhadra, 04009 Almería, Spain; mvicsan150@g.educaand.es (M.F.V.-S.); salodel551@g.educaand.es (S.H.A.-D.); jrosjim793@g.educaand.es (J.J.R.-J.); mtorroj146@g.educaand.es (M.C.T.-R.); azorloz567@g.educaand.es (A.Z.-L.); msegmor692@g.educaand.es (M.J.S.-M.); 2 Department of Psychology, University of Almería, 04120 Almería, Spain; mm.molero@ual.es (M.d.M.M.-J.); perezfuentes@ual.es (M.d.C.P.-F.)

**Keywords:** distance learning, vocational training, quality of education, academic satisfaction, academic burnout

## Abstract

At a time when distance vocational training is on the rise, it seems logical to investigate the variables that can affect the quality of such teaching. The usability of the virtual environment, as well as the behaviour and disposition of the teaching staff, emerge as key factors that influence burnout, engagement, and academic satisfaction. Using a cross-sectional sample of 208 distance vocational training students, the mediating role of burnout and academic engagement in the relationships established between the usability of the virtual environment, teacher support, and academic satisfaction was analysed. On the other hand, multiple regression analyses were carried out in order to investigate the relationships between the challenges and obstacles faced by distance vocational training students and their level of burnout or engagement. Our results confirm the mediating role of academic burnout and engagement in students’ academic satisfaction. Regression analyses suggest that the obstacles faced by distance vocational education and training (D-VET) students influence their level of academic burnout or engagement. Our findings are consistent with the current understanding of the role that certain variables play in the well-being of students and which, in turn, influence the quality of teaching.

## 1. Introduction

In Spain, vocational education and training (VET) is a type of non-university higher education leading to the award of Higher Vocational Training Technician qualifications, with a MECES 1 level (Spanish Framework of Qualifications for Higher Education), equivalent in the context of the European Union to an EQF 5 level (European Qualification Framework).

The importance given to VET, both in the context of the European Union and in Spain, is a readily apparent fact, both in common European policies and in national policies. At the European level, the Osnabrück Declaration [1] aims to strengthen VET as a mechanism to recover and ensure fair transitions toward digital and green economies. The Osnabrück Declaration [1] focuses on four main objectives for the years 2021–2025, one of them being promoting resilience and excellence through flexible, inclusive, and quality VET. Furthermore, the European Parliament Resolution of 17 December 2020 on the Council Recommendation on vocational education and training (VET) for sustainable competitiveness, social fairness, and resilience (2020/2767(RSP)) [2] supports the European Quality Assurance Reference Framework for Vocational Education and Training (EQAVET) [3]. EQAVET [3] sets out the need to strengthen quality in all VET sectors and for all types of delivery, including practical learning (Dual VET), individualised and digital learning (distance or hybrid learning), and its assessment/recognition/certification, which concerns the quality of learning outcomes, certification and assessment, stakeholder consultation, the role of teachers and trainers, practical learning, flexibility, and digital learning. 

In Spain, as shown in Table 1, the number of students enrolled in distance vocational education and training (D-VET) increased by 48.2% between the academic year 2010–2011 and the academic year 2021–2022, and even more in the region of Andalusia, where there has been a percentage increase of 301.14%. Likewise, during the same period, the number of centres offering D-VET increased by 67.59% in Spain and 730% in Andalusia (Table 2). 

However, despite the recommendations for the European level and the boom of VET in Spain, and especially in D-VET, there has not been a correlation in research. The truth is that research focusing on the quality of VET in Spain and in the European Union as a whole is remarkably scarce. VET has not captured the interest of researchers, who instead focus their efforts on evaluating the quality of e-learning in university education, as reflected in several recent reviews of the literature [4,5,6,7], with a minority of research focusing on VET [8]. However, D-VET has its own idiosyncrasies, and there is a need for VET teachers to conduct their own research throughout their careers to improve, promote, and share their practice with the aim of ensuring quality standards in VET, as set out in the Council Recommendation on Vocational Education and Training (VET) for sustainable competitiveness, social equity, and resilience (2020/2767(RSP)) [2].

### 1.1. The Quality of D-VET Education and Training

So far, there is no unanimity on how to assess the quality of e-learning in higher education [7]. Moreover, all the proposed models are based on or have been designed for the evaluation of e-learning at the university level. D-VET is substantially different from e-learning in university education, and the application of an instrument designed based on a model generated for the university environment is not appropriate unless it is adapted to the specific characteristics of D-VET. For example, in Spain, and specifically in Andalusia, the management of D-VET is centralised by the Department of Educational Development and Vocational Training of the Andalusian Regional Government so that all the centres that offer these courses use the same LMS (learning management system) based on the Moodle platform, and the assessment of students is strongly conditioned by the regulations of the regional education administration so that teachers have little autonomy in configuring the LMS for the presentation of contents, as well as methods for assessing and grading the students. In contrast, universities enjoy greater autonomy in these issues. Although both high-level VET and university education operate on higher education, the underlying concepts are clearly different; VET revolves around the students acquiring practical skills and perhaps less theoretical knowledge [9]; hence, the focus of these teachings is different, and the entry requirements in these studies are different, amongst other aspects. 

From certain theoretical perspectives, the quality of the educational service has been taken as a reference to assess the degree of academic satisfaction in students [10], including aspects such as the attitude and behaviour of the teacher or the usability of the LMS or virtual environment; in fact, satisfaction is a construct necessarily related to the pedagogical practices and infrastructure of the institutions, infrastructure that in the case of distance learning is basically reflected in the LMS or virtual environment. 

The precedents of academic satisfaction are as follows: the usability of the LMS or virtual environment and the attitude or behaviour of the teacher. 

From a perspective focused on students’ psychological well-being, satisfaction in the academic environment can be defined as the psychological well-being and enjoyment perceived by students when they carry out experiences linked to their role as students [11] (Medrano and Pérez, 2010). The importance of students’ academic satisfaction lies in their ability to influence academic performance [12,13] or student retention or dropout [14], among other aspects.

On the other hand, the usability of a system can be understood as a quality attribute that evaluates the ease of use of user interfaces [15], with one of its components being the degree of user satisfaction, which is measured in terms of the degree of pleasantness of the design of the virtual environment. Studies on the usability of the virtual learning environment at university levels are frequent [16,17,18,19,20,21]. However, in D-VET, and specifically in Spain, only two studies have been documented, both carried out in the Balearic Islands, with the aim of assessing perceived usability, either by D-VET teachers [22] or by students [23]. D-VET in Andalusia uses a LMS based on the Moodle platform. The Moodle platform is the most-used LMS for remote D-Vet in Spain [24]. The usability of the said virtual environment has not been evaluated, and there is no evidence of this. Bearing in mind that the assessment of perceived usability represents a factor in the analysis and improvement of the instructional designs of a virtual environment, and given that no evaluation of the virtual environment of VET in Andalusia has been carried out, we consider the following as the objective of this research:

**Objective.**  *To assess the perceived usability by students within the virtual environment of D-VET in Andalusia.*

On the other hand, various research has shown that the presence of an educator and availability in virtual environments is positively related to student well-being [5,6,8,25,26]. In fact, several models and instruments to assess the quality of e-learning include, among other aspects, a teaching dimension that relates to the degree of availability and support provided by the teacher, his or her functions, and teaching presence [27,28,29].

Based on these findings and considering that the relationship between the virtual environment’s usability and students’ academic satisfaction, as well as the relationship between teacher availability and students’ academic satisfaction in VET, has not been assessed, we propose our first two hypotheses.

**H1:**  *The degree of usability of the virtual environment has a direct and positive effect on students’ academic satisfaction.*

**H2:**  *The degree of teacher availability and support has a direct and positive effect on students’ academic satisfaction.*

### 1.2. Mediators of Academic Satisfaction: Academic Engagement and Academic Burnout

Both the availability of teaching staff and the quality of the teacher–student interaction, encouraging student participation, as well as the design of the virtual environment adapted to their abilities, contribute to improving students’ academic engagement. Likewise, systematic reviews by Nortvig et al. [8], Paton et al. [9], and Bagriacik-Yilmaz and Banyard [4] show that both the presence of a high teacher in the online environment and the quality of course design are associated with higher levels of academic engagement and academic satisfaction. Academic engagement is understood as the student’s sense of well-being in the face of a given academic challenge [30]. In turn, high levels of academic engagement are related to high levels of student academic satisfaction in e-learning [6,8,31].

On the other hand, if we understand academic burnout as an antagonist of academic engagement, it is logical to think that it has inverse effects on academic satisfaction. In fact, some research has highlighted the negative relationship between burnout and academic satisfaction [12,32], although this relationship has been less studied than the relationship between academic engagement and satisfaction. Academic burnout is understood as a persistent and detrimental state of mind toward studies consisting of emotional exhaustion, feelings of detachment from academic tasks, and perceptions of low ability or efficacy in relation to academic studies [33]. Likewise, the poor design of the virtual environment as well as a limited teacher–student interaction caused by low teacher availability, could be a trigger for academic burnout when these circumstances are perceived as stressful, and the students perceive themselves as unable to cope with them. In fact, a positive relationship between study obstacles and academic burnout has been observed [12,33].

Considering these findings and given that the relationship between perceived usability, academic engagement, academic burnout, and academic satisfaction has not been studied in the D-VET, we propose the following hypotheses:

**H3a** **and H3b:** *The LMS’s usability and teacher availability have a positive effect on academic engagement, and academic engagement has a positive effect on academic satisfaction.*

**H4a** **and H4b:** *The LMS’s usability and teacher availability have a negative effect on academic burnout, and academic burnout has a negative effect on academic satisfaction.*

**Figure 1:**
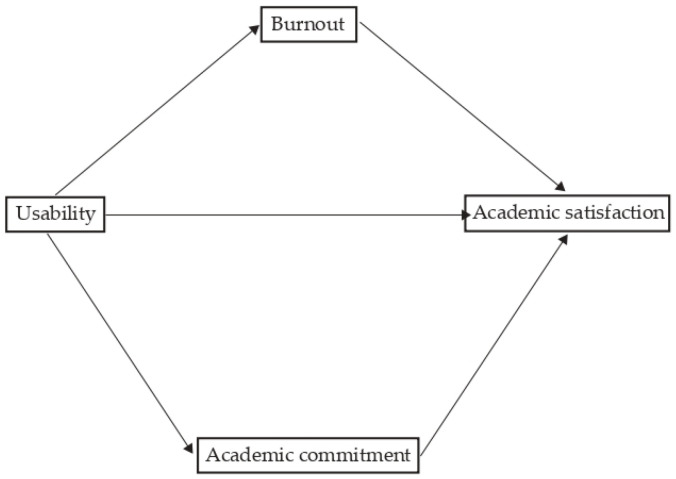
General mediation models: Academic engagement and academic burnout mediate the relationship between the usability of the LMS and academic satisfaction.

**Figure 2:**
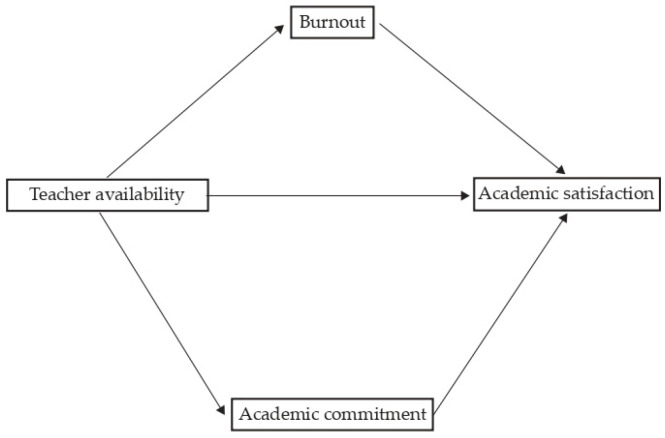
General mediation models: Academic engagement and academic burnout mediate the relationship between teacher availability and academic satisfaction.

**H5:**  *Academic engagement and academic burnout mediate the relationship between the usability of the LMS and academic satisfaction and between teacher availability and academic satisfaction (Figure 1 and Figure 2* *).*

### 1.3. Predictors of Academic Engagement and Academic Burnout: Obstacles and Challenges Faced by Students in D-VET

Students who perceive greater obstacles in relation to their studies experience more burnout and less academic engagement, while higher perceived study facilitators are related to lower levels of burnout and higher levels of academic engagement [12]. A systematic review by Kara et al. [5] identified the challenges and obstacles faced by adult learners in distance learning as related to the management of family life, education, and work, to the learning itself (lack of prior knowledge, lack of concentration for study, etc.), to technical difficulties (insufficient computer skills), to the behaviour of the teaching staff or to the materials and difficulty of the course. It is logical to think that the perception of these circumstances as difficult to cope with determines the appearance of burnout syndrome. In fact, from the perspective of the theory of job demands and resources [34], job demands and resources have direct and indirect effects on work stress and motivation, and this assertion is extensible to the academic environment [35,36]. Thus, students who perceive greater demands (obstacles) and fewer personal resources to cope with them develop higher levels of burnout [36]. Given that academic demands are generally the main predictors of academic burnout [36], the obstacles and challenges faced by distance learners in the online mode could precede the occurrence of burnout, i.e., they precede burnout and, therefore, these challenges (which are ultimately personal circumstances) could be considered as a predictor variable of burnout [37].

Based on the above evidence and with the aim of further exploring the triggers of academic burnout and engagement in D-VET students, we propose hypothesis number 6:

**H6:**  *Perceived obstacles of VET students predict levels of burnout and academic engagement.*

## 2. Materials and Methods

### 2.1. Procedure

The sample was obtained by means of convenience sampling. The inclusion criteria were that participants were of legal age and that they were studying higher vocational training in Andalusia at the time of completing the questionnaire. The format of the questionnaires was online. Participants were contacted through the same virtual platform they used for their studies. Participants were informed of the aims of the study and the anonymity of their responses. After expressing their consent to participate in the research, participants completed the questionnaire individually. Participation was voluntary, and no rewards were given for taking part in the research.

### 2.2. Participants

The sample consisted of 208 students (*n_females_* = 173; 84.1%) who were studying D-VET courses in the Higher-Level Training Cycle (HLTC) (In Spanish: Grado Formativo de Grado Superior) and in 10 vocational training centres in Andalusia, aged between 19 and 63 years (M = 38.24; SD = 9.07). At the time of answering the questionnaire, 65.4% declared to be working (Table 3).

### 2.3. Instruments

In order to achieve our objective and test our hypotheses, the participants in our sample answered a questionnaire with a series of questions related to the usability of the virtual environment, the behaviour and availability of the teaching staff, academic commitment, academic burnout, academic satisfaction and the challenges they faced as students in the e-learning modality. The questionnaire also included a series of questions related to socio-demographic aspects, such as age, gender, studies being undertaken, etc.

The System Usability Scale-A (SUS-A) [38] was used to measure the usability of the virtual environment. The System Usability Scale-A (SUS-A) is a simple ten-item scale that provides an overview of subjective evaluations on usability. This scale is composed of several items that cover a variety of aspects of system usability, such as the need for support, training, and complexity, and, therefore, have a high level of face validity for measuring the usability of a system [38]. It is a Likert-type scale consisting of 10 forced-choice items in which a statement is made, and the respondent indicates his or her degree of agreement or disagreement with the statement on a 5-point scale, with 1 being strongly disagree and 5 being strongly agree. The scale was adapted by substituting the word system for the Moodle platform in each statement (e.g., “I think the Moodle platform is easy to use”). Cronbach’s alpha coefficient was high (α = 0.85). In order to assess the degree of usability, we took the adjectival ratings established by Bangor et al. as a reference [39,40] so that a virtual environment with a score of less than 50 were considered unacceptable (poor), while scores above 70 were considered acceptable (good).

Teacher behaviour and availability were measured using the Teacher Support Scale of the Distance Education Learning Environments Survey (DELES) [41] in its Spanish version [28]. The scale is composed of 8 statements with which the respondent must show his/her degree of agreement or disagreement on a 5-point Likert-type scale, with 1 being strongly disagree and 5 being strongly agree. This scale is intended to measure students’ subjective perception of teachers’ functions, such as clarifying doubts (e.g., “The teacher answers my questions quickly”), facilitating an understanding of the subject (e.g., “The teacher helps me to identify difficulties in the subject of study”), providing feedback on tasks (e.g., “The teacher provides me with valuable feedback (information) on the completion of my tasks”)” among others. Cronbach’s alpha coefficient was high (α = 0.94).

The Academic Engagement Scale in its abbreviated version (UWES-S) [42] was used to study academic engagement, adapted to Spanish by Parra and Pérez [30]. It is a scale in a self-infographic format composed of 9 items grouped into 3 subscales (vigour, dedication, and absorption) with 3 items each. All items were scored on a 5-point Likert-type scale, with 1 strongly disagreeing and 5 strongly agreeing. The vigour subscale integrated questions in relation to the energy and mental stamina needed to perform a task (e.g., “When I study or do homework, I feel that I am full of energy”), the dedication subscale contained questions in relation to the student’s level of involvement in their studies (e.g., “*I am enthusiastic about my studies*”) and the absorption subscale consists of questions related to the state of concentration and immersion in a task (e.g., “*I am immersed in my studies*”). The scale was slightly adapted to ensure its appropriateness to the vocational training context (e.g., “*I am proud to be in distance learning*”). Cronbach’s alpha coefficient was high for all three dimensions (α_vigour_ = 0.80; α_dedication_= 0.88; α_absorption_ = 0.81).

An adapted and modified version of the Maslach Burnout Inventory (MBI-SS) [43] was applied for use with students. The MBI-SS consists of 16 items that constitute 3 scales: exhaustion (5 items, e.g., “*I feel emotionally drained by my studies*”), which assesses the drain on emotional resources; cynicism (5 items, e.g., “*I doubt the importance of my studies*”), which reflects a cold attitude of detachment; and efficacy (6 items, e.g., “*In my opinion, I am a good student*”), which denotes a lack of competence in studies. All items were rated on a 5-point scale, with 1 strongly disagreeing and 5 strongly agreeing. High scores on burnout and cynicism and low scores on efficacy were indicative of burnout, i.e., all items on the efficacy scale had an inverse score. Cronbach’s alpha coefficient on each of the dimensions was high (α_vigour_ = 0.80; α_dedication_ = 0.88; α_absorption_ = 0.81).

Academic satisfaction was measured using the Academic Satisfaction Scale (ESA) [44]. In this research, the version adapted to Spanish by Vergara-Morales et al. [10] was used. This scale is composed of 7 items that constitute a single factor measuring students’ perceived well-being and enjoyment of their role as learners. The items were modified by replacing the word ‘subject’ with ‘Distance Training Cycle’, as our objectives related to D-VET studies as a whole and not to a specific subject (VET module). This modification was made without changing the meaning of the items, which were answered on a 5-point Likert scale ranging from strongly disagree (1) to strongly agree (5). Cronbach’s alpha coefficient was α = 0.80.

To assess the obstacles and challenges faced by learners, a questionnaire (Annex I) was constructed by listing and ranking the internal, external, and programme-related challenges faced by adult learners in distance education [5]. The questionnaire consisted of 26 items that were answered on a 5-point Likert scale ranging from strongly disagree (1) to strongly agree (5). The Cronbach’s alpha coefficient was α = 0.80.

### 2.4. Data Analysis

To calculate the overall usability of Moodle’s platform, the procedure established by Brooke [38] was followed. First, the score contributions of each item were summed (the score contribution of each item ranged from 0 to 4). For items 1, 3, 5, 7, and 9, the score contribution was the scale position minus 1. For items 2, 4, 6, 8, and 10, the contribution was 5 minus the scale position. Secondly, the sum of the scores was multiplied by 2.5 to obtain the total usability value. Following this procedure, scale scores ranging from 0 to 100 were obtained. A range of scores were established according to the adjective rating scale established by Bangor et al. [39,40].

Subsequently, descriptive and bivariate correlation analyses were carried out. On the other hand, to assess the predictive power of the challenges faced by VET students in the virtual modality on academic burnout and engagement, a stepwise multiple linear regression analysis was carried out, eliminating non-significant predictor variables from the model. Collinearity diagnostics were also performed.

Finally, two mediation analyses were conducted to test the direct effects between usability, teacher behaviour, and students’ academic satisfaction (H1 and H2), between usability, teacher behaviour, academic engagement, and academic burnout (H3a and H4a), and between academic engagement and academic burnout on academic satisfaction (H3b and H4b), as well as the indirect effects of academic engagement and academic burnout on academic satisfaction (H5). Model 4 of the PROCESS macro for SPSS [45] was used for indirect effects. This macro provides estimates on multiple mediator spillover effects, standard errors (SE), and confidence intervals (CI). The technique used is bootstrapping, with 5000 samples and a non-parametric resampling procedure that does not impose an assumption of normality on the sampling distribution. If the confidence intervals (95% CI) do not contain zero, then the indirect effects are considered statistically significant [45].

All calculations were performed using SPSS software. (IBM SPSS Statistics 22).

## 3. Results

### 3.1. Usability Analysis of the Working Environment

The Moodle platform was rated OK or better, with 82.2% of respondents rating it as good, excellent, or the best imaginable (Table 4). Thus, most students considered the Moodle platform to be OK, good, or excellent.

### 3.2. Correlation Analysis

All variables involved in this study correlated significantly to the hypotheses stated, except for the sub-factors related to technical, domestic, and work-related challenges (Table 5).

### 3.3. Multiple Linear Regression

We estimated a multiple linear regression model using the stepwise entry method to predict the effect of challenges faced by virtual VET students regarding academic burnout and engagement. In all cases, the assumptions of independence, non-collinearity, and homoscedasticity had a VIF below 5 and a tolerance index above 0.2.

The results confirm that the level of burnout in virtual VET students can be predicted by institutional, learning, and management challenges [y = 0.688 + 0.25*(institutional challenges) + 0.286*(learning challenges) + 0.108*(management challenges)], excluding from the equation the rest of the variables, i.e., those related to technical difficulties, work and homework-related difficulties and the tutor (Table 6). Similarly, it was confirmed that from the challenges and difficulties that relate to learning, with the institution and the tutor, it is possible to predict the level of academic engagement in students through the following equation: y = 4.897 + −0.279*(learning challenges) + −0.171*(institutional challenges) + −0.105*(tutor challenges) (Table 7).

### 3.4. Mediation Analysis

In order to determine whether burnout and academic satisfaction act as mediating variables in the relationships between the usability of the LMS, teacher support, and academic satisfaction, two mediation analyses (model 4) were run with the PROCESS macro for SPSS [46]. The bootstrapping technique with 5000 subsamples was used to estimate the confidence interval (95%).

As shown in Table 8, the results regarding the mediating effect between academic burnout and engagement on the relationship between the usability of the LMS and academic satisfaction did not reveal a significant direct effect between usability and academic satisfaction (β = 0.0017, SE = 0.0019, *p* = 0.3774; 95%, CI [−0.0021–0.0054]). The effects of the LMS’s usability on the mediating variables were significant in both cases (burnout: β = −0.0142, SE = 0.0024, *p* < 0.001; 95%, CI [−0.0189–−0.0096]; academic engagement: β = 0.0132, SE = 0.0026, *p* < 0.001; 95%, CI [0.0081–0.0183]). In the same way, the effects of the mediating variables on academic satisfaction turned out to be significant (burnout: β = −0.05089, SE = 0.0696, *p* < 0.001; 95%, CI [−0.6461–−0.3716]; academic engagement: β = 0.5317, SE = 0.0638, *p* < 0.001; 95%, CI [0.4059–0.6575]). In the global model analysis, the indirect effect of the LMS’s usability on job satisfaction was found to be significant (burnout: β = 0.0070, SE = 0.0018, *p* < 0.001; 95%, CI [0.0040–0.0109]; academic engagement: β = 0.0072, SE = 0.0020, *p* < 0.001; 95%, CI [0.0033–0.0110]), confirming the mediating effect (total mediation) of academic burnout and academic engagement, which partly ratifies our hypotheses (Table 8).

Additionally, the results revealed a direct and positive effect between teacher support and academic satisfaction (β = 0.0898, SE = 0.0381, *p* < 0.001; 95%, CI [0.2147–0.3649]). The effect of teacher support on the mediating variables was found to be significant (burnout: β = 0.4187, SE = 0.0440, *p* < 0.001; 95%, CI [−0.5054–−0.3319]; academic engagement: β = 0.3677, SE = 0.0502, *p* < 0.001; 95%, CI [0.2687–0.4668]), as well as the effects of mediating variables on academic satisfaction (burnout: β = 0.3436, SE = 0.0645, *p* < 0.001; 95%, CI [−0.4707–−0.2164]; academic engagement: β = 0.4947, SE = 0.0565, *p* < *0*.001; 95%, CI [0.3834–0.6061]). In the analysis of the overall model, the indirect effect of teacher support on academic satisfaction also appeared significant, so we can speak of partial mediation (burnout: β = 0.1438, SE = 0.0314, *p* < 0.001; 95%, CI [0.0865–0.2096]; academic engagement: β = 0.1819, SE = 0.0373, *p* < 0.001; 95%, CI [0.1125–0.2581]) (Table 9).

Thus, the results of the mediation analysis, both fully and partially, confirm our hypotheses: both burnout and academic engagement act as mediators in the relationship established between the LMS’s usability, teacher support, and academic satisfaction.

## 4. Discussion

The general objective of this research was to determine certain antecedents and mediators of the academic satisfaction of vocational training students in the distant learning mode. To this end, the usability of the LMS, academic commitment, and satisfaction, certain variables external to the teaching–learning process itself (challenges, difficulties and obstacles related to the reconciliation of personal and family life and study), as well as academic satisfaction itself, were assessed.

Our first objective was to assess the usability of the LMS as perceived by D-VET students in Andalusia. Most students (82.2%) considered the Moodle platform to be adequate, while 17.8% rated it as poor, horrible, or the worst imaginable, but our data do not allow us to determine which aspects of the LMS were taken into account to rate the LMS in one sense or the other. To our knowledge, this is the first evaluation of the usability of the D-VET Moodle platform in Andalusia from the student’s perspective. Similar research has been carried out in the Balearic Islands [23], although using the PSSUQ questionnaire (Post Study System Usability Questionnaire) [47], which reached similar results. However, in the research carried out by Lirola-Sabater and Pérez-Garcias [23], the usability variable was not related to any other variable, while, in our study, the data showed an indirect (mediated) relationship with academic satisfaction. Lirola-Sabater and Pérez-Garcias [23] stated that errors in the design of the LMS or its own complexity could lead to situations where students do not achieve the objectives of the subject or even drop out of their studies. Our data reveal the relationship between usability and academic satisfaction, with academic satisfaction being a powerful predictor of academic performance [12,13] and student retention or dropout [14]. On the other hand, most research on the usability of the virtual environment has been conducted in university studies [16,17,20,21]. While Fernández-Coca et al. [16] and Katsanos et al. [17] are limited to assessing the usability of a given LMS and/or demonstrating the validity and reliability of the instrument, Orfanou et al. [20] and Revythi and Tselios [21] focused on determining which prior aspects (perceived self-efficacy, intention to use, attitude towards the Internet as a learning tool) could influence the usability score. However, none of this research reveals the possible consequences of the LMS usability rating, which is the focus of our research. Thus, our results reveal a positive relationship between the usability of the virtual environment and students’ academic satisfaction. This, however, is not a direct relationship, as we predicted in our hypothesis number 1, but an indirect relationship mediated by academic burnout and engagement. In any case, higher LMS usability scores translate into higher levels of academic satisfaction, which partly confirms our first hypothesis. Recent studies [48] show that the quality of online course design directly influences students’ academic satisfaction; however, our results indicate that this relationship could be one that is mediated by burnout and academic commitment.

Another aspect that, according to previous research, can determine the degree of academic satisfaction in VET students is the degree of availability and support from the teaching staff. We hypothesised that teacher support has a direct and positive effect on students’ academic satisfaction, and our results confirmed this. Thus, the fact that the teacher responds quickly to the doubts raised by the students provides useful feedback for the completion of tasks, encourages active participation, and, in short, is easily accessible, directly increases the satisfaction of students, which fully confirms our hypothesis number 2. What is certain is that the presence and availability of the teacher in virtual environments favours students’ well-being [5,6,8,25,26], and this fact is accepted from a theoretical point of view [27,28,29]. Muzammil et al. [6] established a relationship between the quality of the teacher–student interaction and academic satisfaction through academic engagement in distance university studies. In our case, in addition to this indirect relationship, we also found a direct effect of one variable on the other. Other qualitative studies carried out in Finland and New Zealand with VET students [25,26] highlighted the need to establish an effective teacher–student relationship that is capable of favouring student learning and participation; however, this need is based on theoretical aspects and statistically unproven assertions, issues to which we have made contributions from a quantitative point of view. These results agree with some of the main hypotheses of the academic satisfaction model [49], evidencing the importance of some variables in the academic experience of students who study on platforms mediated by technology. In this sense, our research incorporates some of the variables not included in the study carried out by Zalazar-Jaime et al. [50], such as students’ need to interact with teachers, computational self-efficacy, and academic commitment. Therefore, our results complement this research.

Hence, our results shed light on the relationships between the usability of the LMS, teacher availability, academic engagement, and academic satisfaction. While the usability of the LMS has a direct and positive effect on academic satisfaction, both the direct and indirect effects of teacher support on academic satisfaction are also significant. Thus, both the usability of the LMS and teacher support have a positive effect on academic engagement and satisfaction, as previously evidenced [6], although only in the case of academic engagement, which fully confirms our hypotheses H3a and H3b. This implies that higher scores on the system’s usability and teacher support lead to higher student academic engagement, which translates into higher levels of study energy, engagement, and concentration, and in turn, leads to higher academic satisfaction. Gray and DiLoreto [31] demonstrated the mediating role of academic engagement in the relationship between teacher presence and academic satisfaction in a sample of distance-learning university students using structural equation modelling. We found similar results using the macro process for SPSS [46], and we incorporated another negative variable that enriched the model, academic burnout, which is understood by some authors as an antagonist of academic engagement [12,32].

Therefore, we predict that both the of usability LMS and teacher support may be inversely related to academic burnout and that burnout may have negative effects on academic satisfaction. Our results confirm our hypotheses H4a and H4b. Thus, perceiving the LMS as inadequate with poor teacher support could generate higher levels of burnout, which translates into exhaustion in coping with studies, emotional exhaustion, and feelings of ineffectiveness and detachment from academic tasks. In turn, higher levels of burnout trigger lower levels of academic satisfaction. The study conducted by Supervía and Bordás [32] shows the negative influence of academic burnout on academic satisfaction in compulsory secondary education students, while the research conducted by Martínez-Martínez et al. [12] with a sample of university students (conventional face-to-face studies) shows the effects of both academic engagement and academic burnout on future academic success. Our research is the first to be carried out in the context of VET, in which specific aspects of this type of education are related to academic satisfaction.

In short, our data reveal that both academic engagement and academic burnout act as mediators of academic satisfaction, whether this dependent variable is the usability of the virtual environment or the support and behaviour of the teaching staff, which fully confirms our hypothesis number 5 (H5). However, both academic burnout and academic engagement are affected by other variables, internal or external to the D-VET teaching process itself. We can recall that distance education, particularly vocational education and training, presents certain challenging characteristics given that students often have competing work and family demands on their study time. Based on the research conducted by Kara et al. [5], we predict that certain challenges or obstacles perceived by VET students correlate directly with both levels of burnout and academic engagement. Our hypothesis number 6 (H6) was partially fulfilled, as it was not possible to include all challenges in a single model to predict either burnout or academic engagement. Learning-related barriers (lack of interest, lack of prior knowledge, low concentration on the study, etc.) and those related to the institution (inadequate learning materials, too demanding programme, lack of institutional support) were good predictors of both academic burnout and academic engagement, implying that high levels of both variables are related to burnout and lower levels can predict academic engagement. On the other hand, management challenges (the inability to balance study, work, family, and social life) seem to be a good predictor of academic burnout, while teacher–student and student–student interactions seem to be key variables in the development of academic engagement. In the research carried out by Martínez-Martínez et al. [12], certain obstacles and facilitators of university students’ well-being were analysed; however, these aspects did not include variables external to the university itself that could also be related to this well-being, such as difficulties in family management or work, variables that have been included in this study, which is the first research to assess these issues in distance education and specifically in D-VET.

In a systematic review of the literature [51] carried out recently, the critical success factors of LMSs in VET institutions were identified, distinguishing the following two groups of factors: human and technological. Our results could easily be framed within this model, enriching it with different sub-factors in order to create an extensive model for the much better use of the LMS in D-VET.

### 4.1. Limitations and Future Lines of Research

Our research has certain limitations that must be taken into account when interpreting the results. Given that 17.3% of the students surveyed rated the Moodle platform as inadequate, it is necessary to establish the reasons that led them to rate it as such. This requires qualitative research to identify those aspects of the LMS that need to be improved, such as the complexity of the interface, the organisation of information or its universal design, and the information implemented in the course itself.

Another limitation is related to the use of self-report measures, which could be influenced by students’ attitudes toward the Internet or new technologies in general as learning tools and their previous experience in this or other types of distance learning, among other aspects. We believe that future research should be complemented by feedback from teaching staff and other actors involved in distance learning (coordinators and supervisory staff), including external auditors, in order to verify the consistency of the data provided.

The sample used in our research could constitute another weakness to our research, given that it does not include the entire catalogue of degrees taught in the distance learning modality in Andalusia. Replicating this research with a larger sample, selected through probabilistic sampling and made up of students from other professional families, could serve to corroborate our conclusions.

The cross-sectional nature of our research design is another limitation of this study. In order to confirm the causal relationships hypothesised in this research, it is desirable to carry out studies of a longitudinal nature in order to verify the changes associated with the passage of time.

It should be noted that our research does not start from a defined, let alone contrasted, theoretical model because such a model has not yet been proposed for VETaD. Nevertheless, we believe that this research and its findings, despite the limitations described above, open relevant avenues for future research into the quality of D-VET teaching. Thus, we believe that it would be appropriate to adapt some of the models proposed for university education [27,28,29] to distance vocational education and training.

### 4.2. Practical Implications

The results of our study suggest that both the usability of the virtual environment and the practices carried out by teachers are key factors in the development of academic engagement, academic burnout, and academic satisfaction. Therefore, in order to improve academic satisfaction (which could have an impact on performance and lower dropout rates), it is desirable to consider interventions to optimise the design of the virtual environment and teaching practices. In this sense, in order to improve the teacher–student relationship in virtual environments, Ryökkynen et al. [25] suggested the need to emphasise other dimensions of the teaching profession than subject knowledge, such as guidance that stimulates students’ self-direction, strengthening the construction of meaning and carrying out a process of reflection. Needless to say, for it to be possible to invest in a teacher–student dialogue that helps students develop their skills and competencies, adequate time must be made available for this, so an adequate teacher–student ratio is seen as a prerequisite.

On the other hand, given that certain aspects prior to the teaching–learning process, such as work, family, and social demands, have an impact on the levels of academic burnout, it is advisable to implement actions to identify early those students who might need help, actions that consist of prior counselling in order to guide students, for example, on the number of modules (subjects) recommended to be taken in a specific time. Similarly, preparatory courses could be designed to advise students on the technical requirements for this type of education, including training related to the use of virtual teaching platforms or self-directed learning and courses that are already offered in institutions with extensive experience in distance university education, such as the Spanish National University of Distance Education (Universidad Nacional de Educación a Distancia).

## 5. Conclusions

In a context in which national and European policies are committed to vocational training, which has resulted in an increase in both the number of students and the number of centres offering these courses and given the rise of D-VET and its particularities, it seems logical to implement practices that favour quality D-VET, in pursuit of better training for students and a lower dropout rate. Academic satisfaction emerges as a key variable for achieving these goals, while the design of the virtual environment or LMS, as well as the teaching staff’s practices in the online environment, seem to be fundamental aspects to take into account in order to achieve the desired student satisfaction, either through their direct influence on satisfaction or through indirect effects via academic commitment and burnout.

In general terms, our research shows the importance of certain variables in the academic experience of students who study on technology-mediated platforms. Thus, our findings can serve as a management model to improve the user experience, carrying out actions that aim to improve the quality of the LMS, the quality of attention from D-VET teachers, and academic guidance prior to admission in this type of education. We believe that this last aspect is very important because it would be good to reinforce the computer skills of users and their personal resources for managing family and professional life. Based on our findings, it is possible to design D-VET programs that provide more satisfying academic experiences, increasing achievement, perseverance, and academic success.

## Figures and Tables

**Table 1:** Number of students enrolled in distance or face-to-face VET in Andalusia and Spain.

Academic Year	Students Enrolled in Face-to-Face Higher-Level Vocational Training		Students Enrolled in Distance Higher-Level Vocational Training		Total (Face-to-Face Plus Distance)	
	Spain	Andalusia	Spain	Andalusia	Spain	Andalusia
2010–2011	274.259	44.549	22.568	4.018	296.827	48.567
2012–2013	294.067	47.922	28.231	4.817	322.298	52.739
2013–2014	308.614	49.868	33.035	4.527	341.649	54.395
2014–2015	319.305	51.863	37.215	5.092	356.520	56.955
2015–2016	308.822	52.437	39.313	5.831	348.135	58.268
2016–2017	295.759	49.059	41.842	5.584	337.601	54.643
2017–2018	313.461	52.886	47.424	6.503	360.885	59.389
2018–2019	353,235	62,378	59.934	7.379	413.169	69.757
2019–2020	370.159	67.511	76.547	10.073	446.706	77.584
2020–2021	405.915	75.358	101.420	12.406	507.335	87.764
2021–2022	406.441	75.245	125.423	16.118	531.864	91.363

Source: Non-university Education Statistics. General Subdirectorate for Statistics and Studies of the Ministry of Education, Culture and Sport.

**Table 2:** Number of institutions offering higher level vocational training as distance or face-to-face VET in Andalusia and Spain.

Academic Year	Institutions Offering Face-to-Face Higher Level Vocational Training		Institutions that Offer Distance Higher Level Vocational Training		Total (Face-to-Face Plus Distance)	
	Spain	Andalusia	Spain	Andalusia	Spain	Andalusia
2010–2011	1583	330	91	10	1674	340
2012–2013	2195	434	161	29	2356	463
2013–2014	2236	438	213	31	2449	469
2014–2015	2285	448	239	38	2524	486
2015–2016	2310	459	263	43	2573	502
2016–2017	2388	483	287	48	2675	531
2017–2018	2441	510	299	50	2740	560
2018–2019	2477	517	321	51	2798	568
2019–2020	2537	549	354	65	2891	614
2020–2021	2592	557	406	74	2998	631
2021–2022	2653	579	453	83	3106	662

Source: Non-university Education Statistics. General Subdirectorate for Statistics and Studies of the Ministry of Education, Culture and Sport.

**Table 3:** Sociodemographic data.

	Frequency	%
HLTC currently studying	208	100
Initial D-VET in HLTC(Travel Agencies and Event Management)	10	4.8
Initial D-VET in HLTC (Early Childhood Education)	58	27.9
Initial D-VET in HLTC (Tourist Accommodation Management)	11	5.3
Initial D-VET in HLTC (Sales Management and Commercial Spaces)	3	1.4
Initial D-VET in HLTC (Tourist Guide, Information and Assistance)	3	1.4
Initial D-VET in HLTC (Social Integration)	100	48.1
Initial D-VET in HLTC(Transport and Logistics)	23	11.1
Employment status	208	100
Housewife/Househusband	7	3.4
Unemployed (not seeking employment)	15	7.2
Unemployed (seeking employment)	47	22.6
Retiree	3	1.4
Worker	136	65.4

**Table 4:** Usability analysis of the virtual environment.

Rating	Frequency	%
Worst imaginable	3	1.4
Awful	10	4.8
Poor	24	11.5
Ok	60	28.8
Good	43	20.7
Excellent	57	27.4
Best imaginable	11	5.3
Total	208	100

Adjectival ratings: frequency and percentage.

**Table 5:** Descriptive statistics and correlations of this study * *p* < 0.05; ** *p* < 0.01.

	*M*	*SD*	1	2	3	4	5	6	7	8	9	10	11	12	13	14	15	16	17	18
1.Usability	70.49	20.62	1																	
2. Academic Satisfaction	3.85	0.92	0.35 **	1																
3. Burnout	2.26	0.75	−0.38 **	−0.76 **	1															
4. Exhaustion	2.67	1.09	−0.31 **	−0.56 **	0.83 **	1														
5. Cynicism	2.00	1.11	−0.27 **	−0.67 **	0.86 **	0.55 **	1													
6. Professional efficiency	3.87	0.65	0.36 **	0.57 **	−0.61 **	−0.29 **	−0.38 **	1												
7. Academic Commitment	2.68	0.81	0.33 **	0.77 **	−0.71 **	−0.54 **	−0.60 **	0.57 **	1											
8. Vigour	3.26	0.93	0.23 **	0.56 **	−0.62 **	−0.51 **	−0.50 **	0.45 **	0.88 **	1										
9. Dedication	4.16	0.90	0.35 **	0.81 **	−0.67 **	−0.51 **	−0.61 **	0.46 **	0.85 **	0.59 **	1									
10. Absorption	3.62	0.90	0.32 **	0.69 **	−0.61 **	−0.41 **	−0.49 **	0.59 **	0.91 **	0.74 **	0.69 **	1								
11. Teacher Support	3.76	1.00	0.31 **	0.66 **	−0.55 **	−0.46 **	−0.51 **	0.27 **	0.45 **	0.36 **	0.47 **	0.37 **	1							
12. Challenges	2.36	0.66	−0.41 **	−0.48 **	0.59 **	0.54 **	0.43 **	−0.40 **	−0.47 **	−0.42 **	−0.35 **	−0.47 **	−0.46 **	1						
13. Management D.	3.04	1.18	−0.32 **	−0.35 **	0.52 **	0.52 **	0.35 **	−0.35 **	−0.39 **	−0.34 **	−0.30 **	−0.38 **	−0.28 **	0.76 **	1					
14. Learning D.	2.05	0.87	−0.38 **	−0.47 **	0.62 **	0.49 **	0.48 **	−0.52 **	−0.48 **	−0.44 **	−0.34 **	−0.48 **	−0.37 **	0.71 **	0.56 **	1				
15. Technical D.	1.67	0.86	−0.13	−0.05	0.09	0.11	−0.01	−0.16 *	−0.07	−0.07	<0.001	−0.10	0.02	0.43 **	0.16 *	0.22 *	1			
16. D. of Work	2.72	1.11	−0.07	−0.13	0.14 *	0.12	0.09	−0.14 *	−0.19 **	−0.21 **	−0.08	−0.23 *	−0.07	0.61 **	0.41 **	0.19 **	0.23 **	1		
17. D. domestic	1.74	0.85	−0.32 **	−0.05	0.18 **	0.19 **	0.13 *	−0.09	−0.10	−0.126	0.00	−0.16 *	−0.17 *	0.63 **	0.40 **	0.37 **	0.23 **	0.40 **	1	
18. D. guardian	2.71	1.09	−0.24	−0.40 **	0.39 **	0.34 **	0.32 **	−0.25 **	−0.36 **	−0.32 **	−0.27 **	−0.36 **	−0.44 **	0.68 **	0.36 **	0.39 **	0.19 **	0.32 **	0.34 **	1
19.D. institutional	2.60	1.16	−0.38	−0.60 **	0.61 **	0.60 **	0.51 **	−0.30 **	−0.45 **	−0.35 **	−0.48 **	−0.36	−0.68 **	0.65 **	0.44 **	0.50 **	0.07	0.15 *	0.23 **	0.41 **

**Table 6:** Multiple linear regression model for the effect of challenges on academic burnout.

Predictable Variables	*F (_gl_)*	*R* ^2^	*β*	*SE*	*p*
Model 1	*136.57* *(_1206_)*	*0.631*	*1.193*	*0.101*	*<0.001*
Institutional challenges			*0.413*	*0.035*	
Model 2	*111.84* *(_2205_)*	*0.722*	*0.816*	*0.104*	*<0.001*
Learning challenges			*0.353*	*0.049*	
Institutional challenges			*0.279*	*0.037*	
Model 3	*79.85* *(_3204_)*	*0.735*	*0.688*	*0.111*	*<0.001*
Learning challenges			*0.255*	*0.037*	
Institutional challenges			*0.286*	*0.053*	
Management challenges			*0.108*	*0.038*	

**Table 7:** Multiple linear regression model for the effect of challenges on academic engagement.

Predictable Variables	*F (_gl_)*	*R* ^2^	*β*	*SE*	*p*
Model 1	*61.736* *(_1206_)*	*0.231*	*4.596*	*0.0126*	*<0.001*
Learning challenges			*−0.446*	*0.057*	
Model 2	*42.161* *(_2205_)*	*0.291*	*4.984*	*0.135*	*<0.001*
Learning challenges			*−0.312*	*0.063*	
Institutional challenges			*−0.199*	*0.048*	
Model 3	*30.16* *(_3204_)*	*0.307*	*4.984*	*0.149*	*<0.001*
Learning challenges			*−0.279*	*0.064*	
Institutional challenges			*−0.171*	*0.049*	
Challenges related to the guardian			*−0.105*	*0.049*	

**Table 8:** Direct and indirect associations between the usability of the virtual environment, academic satisfaction, academic engagement and burnout.

Mediator	Usability on the Mediator (A)			Mediator on Academic Satisfaction (B)			Usability on Academic Satisfaction (C)			Mediating Effect (AB)		
	β (SE)	95% CI		β (SE)	95% CI		β (SE)	95% CI		β (SE)	95% CI	
		Lower	Upper		Lower	Upper		Lower	Upper		Lower	Upper
Academic commitment	0.0132 (0.0026) *	0.0081	0.0183	0.5317 (0.0638) *	0.4059	0.6575	0.0017 (0.0019)	−0.0021	0.0054	0.0072 (0.0020) *	0.0033	0.0110
Burnout	−0.0142 (0.0024) *	−0.0189	−0.0096	−0.5089 (0.0696) *	−0.6461	−0.3716	0017 (0.0019)	−0.0021	0.0054	0.0070 (0.0018) *	0.0040	0.0109

* *p* < 0.001.

**Table 9:** Direct and indirect associations between teacher support, academic satisfaction, academic engagement and burnout.

Mediator	Teacher Support on the Mediator (A)			Mediator on Academic Satisfaction (B)			Faculty Support on Academic Satisfaction (C)			Mediating Effect (AB)		
	β (SE)	95% CI		β (SE)	95% CI		β (SE)	95% CI		β (SE)	95% CI	
		Lower	Upper		Lower	Upper		Lower	Upper		Lower	Upper
Academic commitment	0.3677 (0.0502) *	0.2687	0.4668	0.4947 (0.0565) *	0.3834	0.6061	0.2898 (0.0381) *	0.2147	0.3649	0.1819 (0373) *	0.1125	0.2581
Burnout	−0.4187 (0.0440) *	−0.5054	−0.3319	−0.3436 (0.0645) *	−0.4707	−0.2164	0.2898 (0.0381) *	0.2147	0.3649	0.1438 (0.0314) *	0.0865	0.2096

* *p* < 0.001.

## Data Availability

The data presented in this study are available upon request from the corresponding author.
